# Cord Blood T Cells Expressing High and Low PKCζ Levels Develop into Cells with a Propensity to Display Th1 and Th9 Cytokine Profiles, Respectively

**DOI:** 10.3390/ijms22094907

**Published:** 2021-05-05

**Authors:** Khalida Perveen, Alex Quach, Andrew McPhee, Susan L. Prescott, Simon C. Barry, Charles S. Hii, Antonio Ferrante

**Affiliations:** 1 Department of Immunopathology, SA Pathology at the Women’s and Children’s Hospital, North Adelaide, SA 5006, Australia; khalida.perveen@adelaide.edu.au (K.P.); Alexander.Quach@sa.gov.au (A.Q.); charles.hii@adelaide.edu.au (C.S.H.); 2 Adelaide School of Medicine and the Robinson Research Institute, University of Adelaide, Adelaide, SA 5005, Australia; simon.barry@adelaide.edu.au; 3 Department of Neonatal Medicine, Women’s and Children’s Hospital, North Adelaide, SA 5006, Australia; Andrew.McPhee@sa.gov.au; 4 School of Paediatrics and Child Health, The University of Western Australia, 35 Stirling Highway, Perth, WA 6009, Australia; Susan.Prescott@telethonkids.org.au; 5 The ORIGINS Project, Telethon Kids Institute and Perth Children’s Hospital, 15 Hospital Avenue, Nedlands, WA 6009, Australia; 6 School of Biological Sciences, University of Adelaide, Adelaide, SA 5005, Australia

**Keywords:** cord blood T cells, T cell maturation, Th1 and Th2 subsets, PKCζ, cytokines, allergy

## Abstract

Low Protein Kinase C zeta (PKCζ) levels in cord blood T cells (CBTC) have been shown to correlate with the development of allergic sensitization in childhood. However, little is known about the mechanisms responsible. We have examined the relationship between the expression of different levels of PKCζ in CBTC and their development into mature T cell cytokine producers that relate to allergy or anti-allergy promoting cells. Maturation of naïve CBTC was initiated with anti-CD3/-CD28 antibodies and recombinant human interleukin-2 (rhIL-2). To stimulate lymphocyte proliferation and cytokine production the cells were treated with Phytohaemagglutinin (PHA) and Phorbol myristate acetate (PMA). Irrespective of the PKCζ levels expressed, immature CBTC showed no difference in lymphocyte proliferation and the production of T helper 2 (Th2) cytokine interleukin-4 (IL-4) and Th1 cytokine, interferon-gamma (IFN-γ), and influenced neither their maturation from CD45RA^+^ to CD45RO^+^ cells nor cell viability/apoptosis. However, upon maturation the low PKCζ expressing cells produced low levels of the Th1 cytokines, IFN-γ, IL-2 and tumour necrosis factor-alpha (TNF), no changes to levels of the Th2 cytokines, IL-4, IL-5 and IL-13, and an increase in the Th9 cytokine, IL-9. Other cytokines, lymphotoxin-α (LT-α), IL-10, IL-17, IL-21, IL-22 and Transforming growth factor-beta (TGF-β) were not significantly different. The findings support the view that low CBTC PKCζ levels relate to the increased risk of developing allergic diseases.

## 1. Introduction

In developed countries, about 40% of children develop allergic sensitization, resulting in symptomatic disease [1]. With similar trends now evident in developing countries, the disease burden is likely to further escalate [2]. There is therefore an increasing need for validation of biomarkers which identify those at risk of developing allergy and approaches to modulation of immune responses away from allergy promoting response. While T helper 1 (Th1) and regulatory T cells play protective roles in allergy, Th2, Th9 and Th22 promote allergy. Th1 lymphocytes produce Interferon-gamma (IFN-γ) while Th2 cells are mainly producers of interleukin 4 (IL-4), IL-5 and IL-13, which is a hallmark of atopy or allergy. IL-4 promotes Th2 development and differentiation [3], immunoglobulin class switch to Immunoglobulin E (IgE) in B cells, and high-affinity IgE receptor (FcεR1) expression on mast cells. IL-5 is imperative in the survival, activation, development, and differentiation of mast cells and eosinophils and increases basophils degranulation. IL-13, which shares some role with IL-4, also regulates immunoglobulin class switch to IgE and promotes eosinophil trafficking to mucosal sites. Th9 cells are the main producers of IL-9, although it is debatable whether these are Th9 per se or Th2 [4,5,6], with similar roles as Th2 cell cytokines, promoting the development of allergic asthma [5,6,7,8,9,10,11,12,13,14,15,16]. This subset develops in the presence of IL-4 and Transforming growth factor-beta (TGF-β) and is inhibited by IFN-γ [17,18,19]. IL-9 increases IgE expression, causing Bronchial Hyperresponsiveness in inhaled methacholine-induced asthma [20,21] and enhanced mucus production by epithelial cells [22]. It is also involved in accumulation, survival, growth, proliferation activation and differentiation of mast cells [9,23,24]. In mast cells, it increases expression of proteases and the FcεRIα [9,25]. IL-9 also plays a role in airway eosinophilia and promotes eosinophil survival and differentiation and IL-5Receptor (IL-5R) expression [21,26,27]. In contrast, the Th1 cytokine IFN-γ is inhibitory toward development of Th2 and Th9 cells [28,29,30].

We have previously demonstrated that cord blood T cells (CBTC) may display low levels of different Protein Kinase C (PKC) isozymes [31,32,33,34,35] which normalizes during maturation [36] but most interesting was that the levels of PKCζ in immature CBTC correlated with the risk of developing allergic diseases [3]. Babies found to have low expression of PKCζ in their CBTC were at high risk of developing allergy [3]. Examination of development of sensitization found that a PKCζ level of <62% could predict allergy development with an accuracy of 71%, sensitivity of 80% and 63% specificity [33,34]. Further support for the importance of PKCζ levels in CBTC as a potential determinant of allergy control comes from in vitro culture models of CBTC maturation. These studies suggest PKCζ levels may influence the maturation of T cells into Th cells with a propensity to produce either Th1 or Th2 cytokines, such that higher levels of PKCζ appear to skew these responses towards Th1 IFN-γ production. In addition, supplementation of women during pregnancy with ω-3 polyunsaturated fatty acids (fish oil) was associated with an increase in expression of PKCζ in CBTC, with associated protection against allergic sensitization in the first year of life [33,34]. 

Despite these findings, we understand very little about factors and pathways that alter naïve CBTC maturation towards Th1 or Th2 or in other subsets, propensity. Our study seeks to further elucidate these pathways to facilitate early detection that may assist interventions to reduce the epidemic rates of allergic disease through primary prevention. Specifically, here we investigated the relationship between naïve CBTC PKCζ levels and their development to Th1 or Th2 cytokine producers and extended this question to other cytokines which protect against or promote allergy, such as IL-9, IL-17 and IL-10. Since previous studies showed an important role for PKCζ in protection against apoptosis in the human T cell line, Jurkat cells [37], our studies extend to determine whether deficient/low levels of PKCζ influence survival of T cells during this maturation.

## 2. Results

### 2.1. The Levels of PKCζ in Immature CBTC Does Not Affect Cytokine Pattern Expressed and Lymphoproliferation

**Figure 1:**
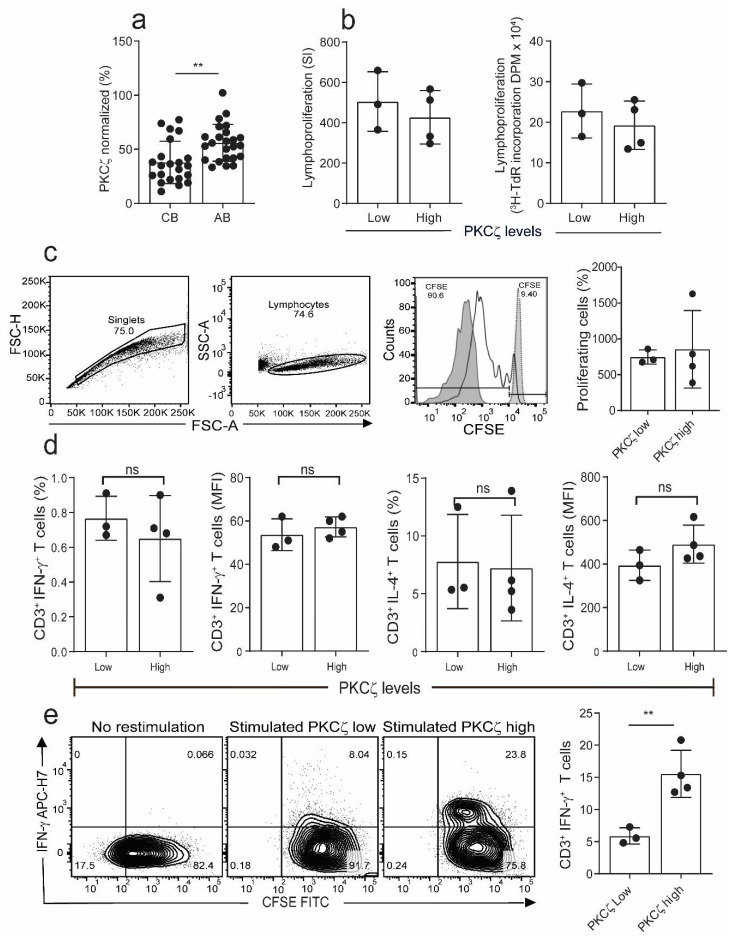
Comparison of proliferation and cytokine production in naïve cord blood T cells (CBTC) in Protein Kinase C zeta (PKCζ) low or high group. (**a**) Comparison of PKCζ between cord blood (CB) and adult blood (AB) T cells, *n* = 24 for each AB or CB. (**b**) Shows lymphoproliferation as a Stimulation index (SI) and disintegrations per minute (DPM) in ^3^H-thymidine pulsed cultures stimulated with Phytohaemagglutinin (PHA) and Phorbol myristate acetate (PMA) (**c**) Purified CBCTs were stained with Carboxyfluorescein succinimidyl ester (CFSE) dye and stimulated with immobilized anti-CD3/-CD28 antibodies for 3 days. Gating and representative histogram for CFSE dilution after exclusion of doublets and dead cells. Overlaid histograms for stained unstimulated and unstained stimulated samples were used as control and for gating the non-proliferating cells and for auto-fluorescence, respectively. (**d**) Naïve CB CD3^+^ T cells were stimulated with PHA/PMA (18 h) and percentage of CD3^+^ T cells producing interleukin-4 (IL-4) and Interferon-gamma (IFN-γ) and median fluorescent intensity (MFI) were examined by flow cytometry assays. (**e**) On day 5 of CFSE stained culture (anti-CD3/-CD28), cells were re-stimulated with PHA/PMA (18 h) for detection of intracellular cytokine. Representative flow dot plots and data for CFSE dye dilution and IFN-γ producing cells in high and low PKCζ group. Data mean ± SD of *n* = 3 for of low and *n* = 4 high PKCζ group. ** *p* < 0.01. ns: not significant (Student’s *t*-test).

In all the following studies, the PKCζ levels were measured by intracellular staining by flow cytometry using fluorochrome-tagged monoclonal antibody. To define T cells expressing low and high PKCζ, we established the 5th (34.6%) percentile from a cohort of normal adult volunteers (Figure 1a). Those expressing PKCζ levels below the 5th percentile were considered to be low and above the cut off as high (Figure 1a). The data also show that there is a significant difference in the level of PKCζ between CBTC and adult blood T cells (Figure 1a).

To examine whether there was any difference in immunocompetence between the low and high PKCζ expressing T cells, we examined the lymphocyte proliferation response in the immature CBTC. The CBTC were stimulated with PHA/PMA, and then the incorporation of tritiated thymidine ([^3^H]-TdR) measured after 72 h of culture. The results showed that the PKCζ low and high cells had comparable ability to proliferate (Figure 1b). Similar results were obtained when proliferation was conducted with CBTC stimulated with anti-CD3/-CD28 antibodies using the Carboxyfluorescein succinimidyl ester (CFSE) assay (Figure 1c). To determine whether the levels of PKCζ expressed by the naïve CBTC (CD45RA^+^/RO^−^) influenced cytokine production, IFN-γ and IL-4, the CBTC expressing either low or high PKCζ were stimulated with Phytohaemagglutinin (PHA) and Phorbol myristate acetate (PMA) for 18h and then intracellular staining was performed to identify IFN-γ and IL-4 producing cells. The data presented in Figure 1d demonstrate that the immature T cells produce much more IL-4 than IFN-γ. There was no difference between low and high expressors. The findings were similar irrespective of whether we used the % of cytokine positive T cells or the Median Fluorescence Intensity (MFI) to run comparisons (Figure 1d). Thus, it is evident that the levels of PKCζ did not affect the predominant IL-4 production by these immature T cells.

When the cells were harvested on day 5 after maturation (anti-CD3/-CD28, without recombinant human interleukin-2 (rhIL-2)) and stimulated with PHA/PMA (18h), those expressing low levels of PKCζ had reduced percentage of IFN-γ producing cells compared to samples expressing high levels of PKCζ (Figure 1e).

### 2.2. CBTC PKCζ Levels and T Cell Survival during Maturation

**Figure 2:**
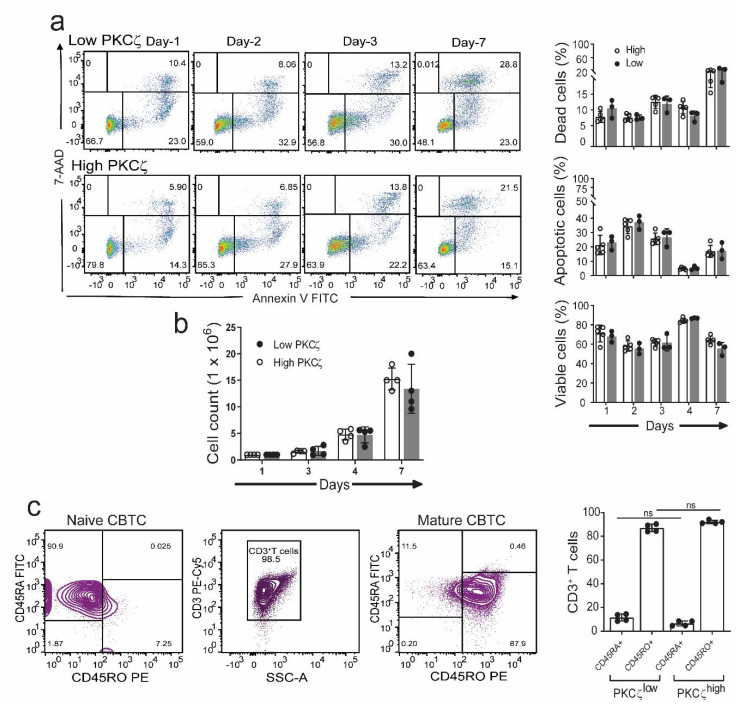
Comparison of viability, apoptosis, and maturation in CBTC expressing low or high levels of PKCζ during maturation in culture. Purified CB CD3^+^ T cells were matured with anti-CD3/-CD28 and recombinant human interleukin-2 (rhIL-2) for 7 days. (**a**) Representative flow dot plots and percentage CB CD3^+^ T cells was analyzed at indicated times and expressed as percentage of dead cells (7-aminoactinomycin D (7-AAD^+^) and apoptotic cells (Annexin V^+^). *n* = 3 for low PKCζ and *n* = 5 for high PKCζ (**b**) Cell counts during maturation assay. (**c**) Representative dot plot for naïve and maturation expression in CB CD3^+^ T cells on day seven. Data mean ± SD of *n* = 4 for low and *n* = 4 high PKCζ group. ns: not significant (**a**,**b**): Student’s *t*-test, (**c**): one-way ANOVA with post hoc Tukey’s multiple comparisons test.

Previous studies showed an important role for PKCζ in protection against apoptosis in the human T cell line, Jurkat cells [37]. It was therefore of interest to see if the low and high PKCζ expressing T cells exhibited different survival rates during their maturation in culture. The purified CBTC were matured by addition of anti-CD3/-CD28 antibodies and rhIL-2. When the cells were analysed for levels of 7-aminoactinomycin D (7-AAD) and Annexin V staining as the indicator of cell death and early apoptosis, respectively, we found no difference in apoptosis and cell survival between the low and high PKCζ CBTC during their 7 day maturation in culture (Figure 2a). The survival levels ranged between 60–85% in the PKCζ high and 55–85% in the PKCζ low samples. Thus, the levels of CBTC PKCζ are unlikely to be controlling the rate of apoptosis or viability. Despite the differences in PKCζ levels, the T cells showed the same level of maturation based on the expression of CD45RO (Figure 2c).

### 2.3. Low PKCζ Expression Is Associated with Decreased Development towards a Th1 Cytokine Profile

**Figure 3:**
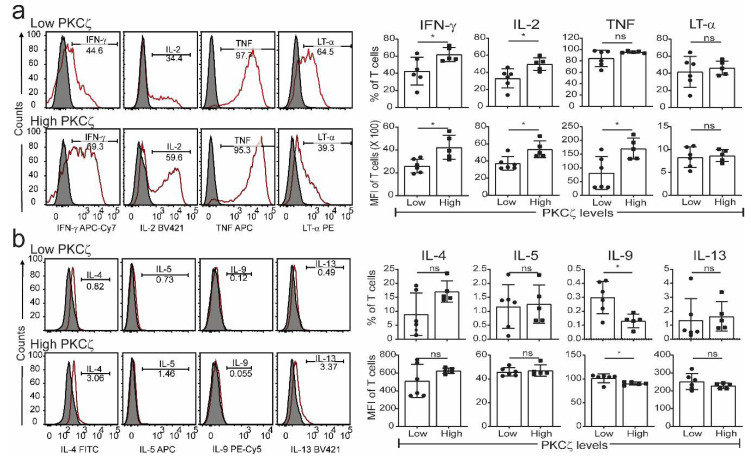
Detection of Th1 and Th2 cytokine in matured PKCζ low and high CBTC. Purified CB CD3^+^ T cells were matured with anti-CD3/-CD28 antibodies with recombinant human interleukin-2 (rhIL-2). On day seven, cells were left unstimulated or stimulated with PHA/PMA overnight. Dead cells were excluded by gating on Fixable Viability Stain 510 (FVS510) negative population and cytokines were analysed by flow cytometry. Representative histograms and graphs for (**a**) Th1 and (**b**) Th2 cytokines in PKCζ low and high samples showing the gating for the analysis of % positive cells for respective cytokines percentage and their MFI. Shaded and empty histograms represent unstimulated and stimulated samples stained with a cytokine-specific antibody. Graphs represent the means ± SD of *n* = 6 for low and 5 for high. * *p* < 0.05. ns: not significant. (Student’s *t*-test).

**Figure 4:**
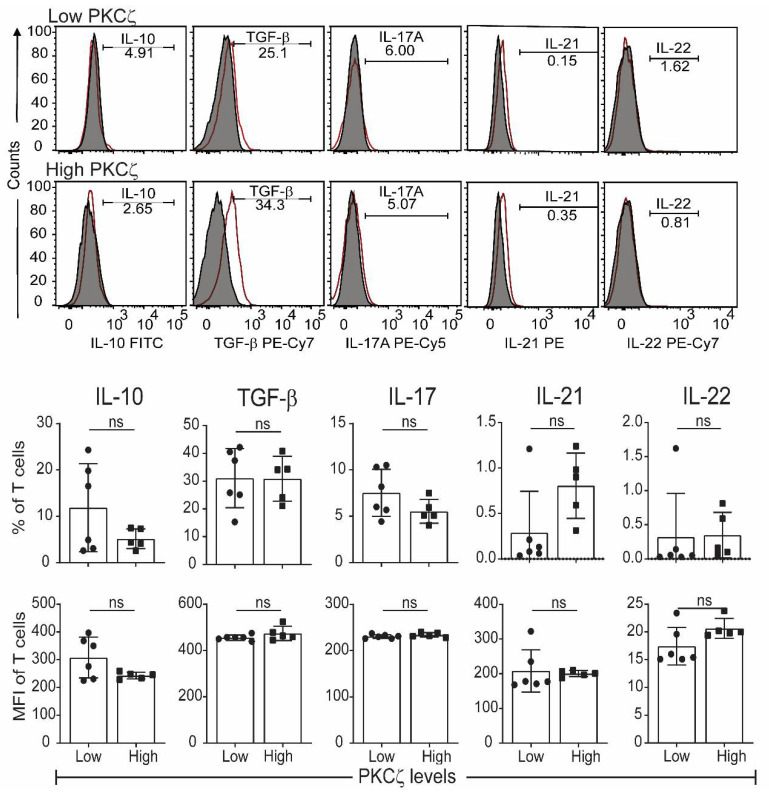
Other T cell cytokines expressed by matured cord blood T cells, which express either low or high levels of PKCζ. Cells were matured by treating with anti-CD3/-CD28 and rhIL-2. After maturation, they were stimulated as per Figure 3. (**a**) Representative histograms for Th or Treg cytokines in PKCζ low and high samples showing the gating for the analysis of graphs for respective cytokine positive cells percentage and their MFI. Shaded and empty histograms represent unstimulated and stimulated samples stained for the respective cytokines. Graphs represent the means ± SD of *n* = 6 for low and 5 for high. ns: not significant. (Student’s *t*-test).

**Figure 5:**
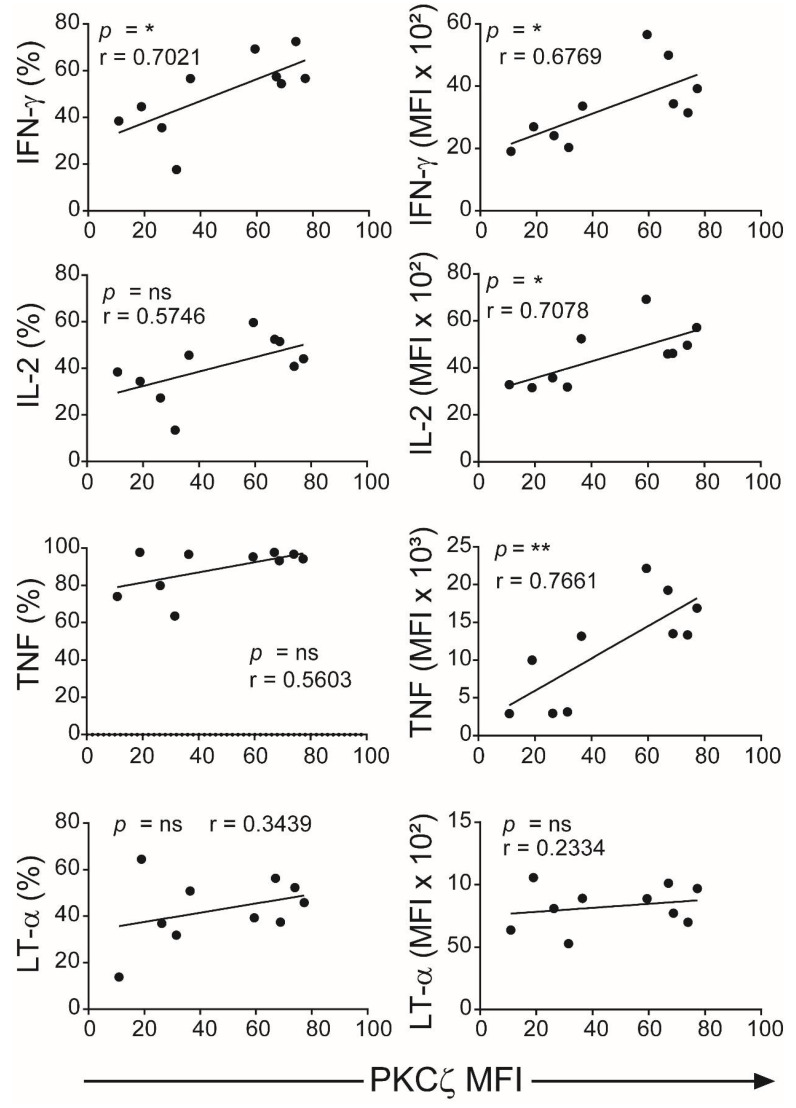
Correlation analyses of levels of PKCζ expression with Th1 cytokines production. Data from Figure 3 were subjected to correlation analysis. The first column represents the Pearson correlation of PKCζ (MFI) with representative cytokines (percentage of CD3^+^ T cells for each cytokine) and the second column represents the MFI of the positive gated cells. * *p* < 0.05, ** *p* < 0.01. ns: not significant. Correlations were performed using the two-tailed Pearson correlation coefficient.

**Figure 6:**
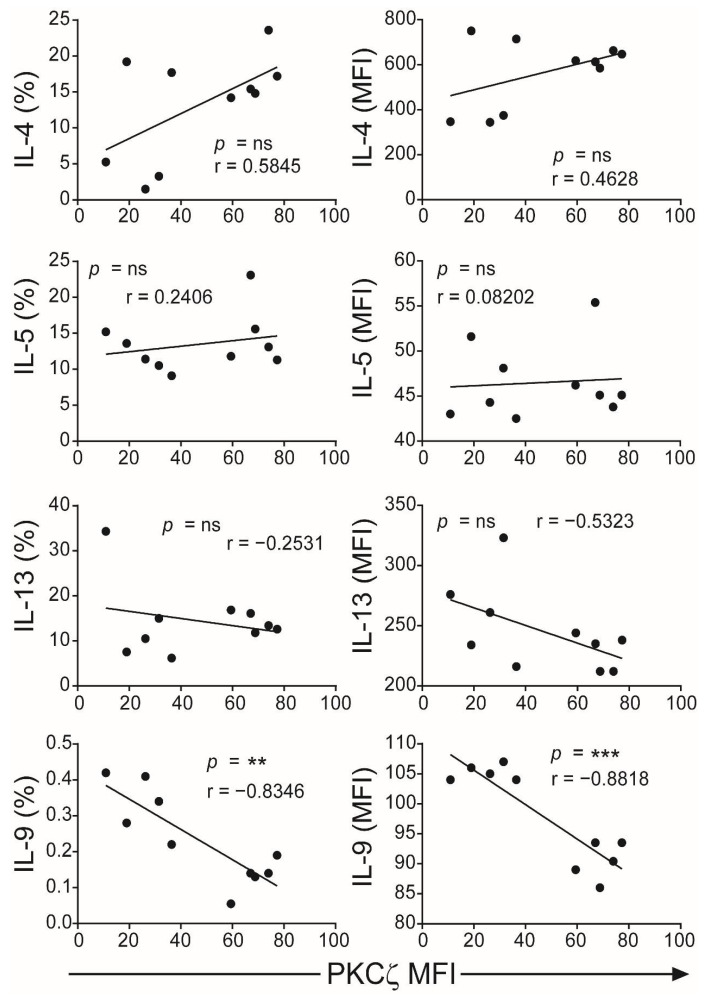
Correlation of PKCζ expression with Th2/Th9 cytokines. Data from Figure 4 were subjected to correlation analysis. The first column represents the Pearson correlation of PKCζ (MFI) with representative cytokines (percentage of CD3^+^ T cells for each cytokine) and the second column represents the MFI of the positive gated cells. ** *p* < 0.01, *** *p* < 0.001. ns: not significant. Correlations were performed using the two-tailed Pearson correlation coefficient.

**Figure 7:**
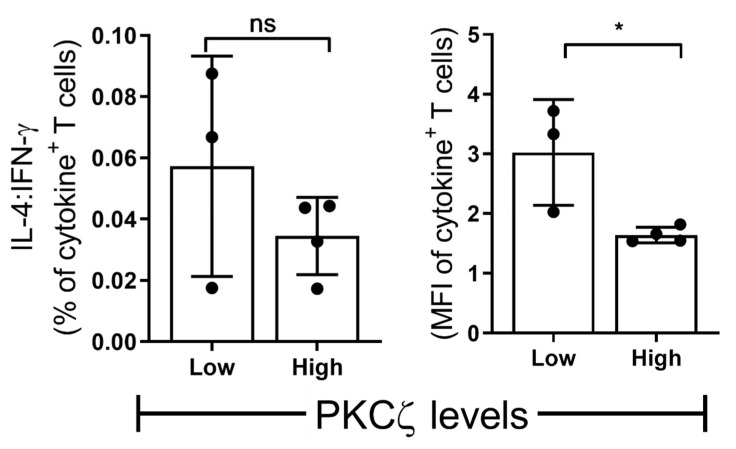
The ratio of Th2 vs. Th1 in low vs. high PKCζ CBTC. Purified CB CD3^+^ T cells were overnight stimulated as in Figure 1 (naive T cells) and Figure 4 and Figure 5 (mature T cells). The cells were gated for total CD3^+^ T cells producing IL-4 and IFN-γ as percentage of T cells or MFI as examined by flow cytometry assays. Comparison of percentage decrease in IL-4: IFN-γ in mature T cells (normalized against naïve T cells) and compared between CB samples expressing low vs. high PKCζ. *n* = 3 for low and *n* = 4 for high PKCζ group. * *p* < 0.05. ns: not significant. (Student’s *t*-test).

The CBTC were matured by adding anti-CD3/-CD28 and rhIL-2 and cultured for 7 days. For assessing the ability to produce cytokines the matured CBTC were stimulated with PHA-PMA. After 18 h the intracellular cytokine expression was determined using fluorochrome tagged monoclonal antibodies. The data showed the expression of lower levels of the Th1 cytokines, IFN-γ, IL-2 and TNF but increased levels of Th9 cytokine IL-9 in the low PKCζ CBTC group compared to the PKCζ high group. In the case of TNF, there was no difference in the % of T cells that expressed TNF between low/high PKCζ expressors but of the cells that expressed TNF, the PKCζ high cells had higher levels of TNF than the low PKCζ cells. Other cytokines, lymphotoxin α (LT-α), IL-10, IL-4, IL-13, IL-5, IL-17, IL-21, IL-22 and TGF-β were not significantly different (Figure 3 and Figure 4). The data were also expressed as correlation analysis between the PKCζ levels and different cytokines. This evaluation confirmed that expression of high levels of PKCζ by CBTC led to the development of T cells with a Th1 skewed response/cytokine production (Figure 5, Figure 6 and Figure S1). Correlation analyses between IFN-γ and other cytokines showed a positive correlation with IL-2, TNF, and LT-α, and an inverse correlation with IL-9 (Figures S2–S4). Thus, as a result of high expression of PKCζ in CBTC, there is skewing towards Th1 cytokine-producing cells, as shown by the ratio of the IL-4:IFN-γ producing cells (Figure 7).

## 3. Discussion

The data demonstrate that when stimulated immature CBTC produce substantially more IL-4 compared to IFN-γ. Previous reports have primarily used CBMC, although a limited number have used purified T cells to examine this question [38,39,40,41]. Our studies confirm and extend these findings by also showing that the deficiency in PKCζ in CBTC does not contribute to the decrease in IFN-γ production and not affect the T lymphocyte proliferation responses of the immature T cells. Examination of T cells during maturation also showed that the decreased expression of PKCζ was not associated with loss of cell viability and apoptosis, although in the Jurkat T cell line PKCζ was found to protect against apoptosis [37].

The different levels of PKCζ expressed in CBTC did not affect either the maturation of the CBTC over the 7-day culture period, i.e., cells progressing from a CD45RA^+^/RO^−^ phenotype to CD45RA^−^/RO^+^ phenotype or T cell growth. The main difference observed was a correlation with skewing the response of the matured T cells—with a propensity to give rise to either a Th1 (in the PKCζ high cells) or Th9 skewed response (in the PKCζ low cells). Examination of matured T cell cytokine responses showed that those derived from low PKCζ expressing CBTC produced lower amounts of Th1 cytokines, IFN-γ, IL-2, TNF but higher Th9 cytokine, IL-9, seen as either a significant increase in the number of cytokine-producing cells and/or in MFI. Pearson correlation analysis of the PKCζ levels against the production of the different cytokines supported the PKCζ level dependency of the Th1 and Th9 responses. This cytokine pattern further supported the finding by showing that there was a strong and significant positive correlation between the levels of IFN-γ expression and each of the other Th1 cytokines, IL-2 and TNF. Although we did not find a correlation with LT-α, this is not surprising since this is fully expressed in immature CBTC. As expected, there was a negative correlation between IFN-γ and the Th9 cytokine, IL-9. However, no significant correlation was found with IL-13, IL-5 and IL-4. However, it was evident that the PKCζ levels in immature CBTC correlated with a decrease in the IL-4:IFN-γ ratio following their maturation. These findings contrast with our previous findings that knocking out PKCζ from CBTC leads to development towards Th2, IL-13 producing phenotype, suggesting that there may be a thresh hold PKCζ level, below which, may promote the Th2 phenotype development [35]. Never-the-less collectively the findings suggest that there may be an intricate network operating in the mechanism of T cell development to acquire specific functional responses. Whether an increase in sample numbers/statistical power modifies the results, remains to be determined.

The results reveal, for the first time, that CBTC expressing high PKCζ levels mature to display Th1 capabilities such as the production of IFN-γ, IL-2 and TNF but low Th9 cytokine IL-9. The levels of PKCζ did not influence the development of cells expressing IL-4, IL-5 or IL-13. Furthermore, CBTC PKCζ levels did not correlate with the development of regulatory cytokines IL-10 and TGF-β producing T cells or cells producing the inflammatory cytokines, IL-17, IL-21 and IL-22. The finding suggests the existence of an influence by the PKCζ levels in naïve CBTC and development of allergen sensitization, due primarily to its influence on the Th1 arm of the immune response and also on Th cells, producing IL-9. Our findings thus suggest that the most likely protective effects of higher expression of PKCζ to allergen sensitization [33] are due to CBTC maturation towards Th1 IFN-γ producing cells with a decrease in Th9, IL-9 producing cells. This supports the view that PKCζ levels measured in CBTC are relevant to allergy risk in later life and that modulating the levels through nutrients such as ω-3 polyunsaturated fatty acids may be of benefit in those at risk of developing allergic diseases.

## 4. Materials and Methods 

### 4.1. Reagents 

The mouse monoclonal IgG_2aκ_ phycoerythrin (PE)-conjugated PKCζ isozyme antibody (clone H-1, and previously validated by our laboratory [32]) was purchased from Santa Cruz Biotechnology (Dallas, TX, USA). The corresponding isotype control was purchased from BD (Franklin Lakes, NJ, USA). RPMI 1640 tissue culture medium (Sigma-Aldrich Cat# R0883), X-VIVO 15 medium (04418Q, Lonza, BSL, Switzerland), foetal calf serum (FCS) and L-glutamine were purchased from SAFC Biosciences (Lenexa, KS, USA). PHA, PMA and human AB serum were from Sigma Aldrich (St. Louis, MI, USA), whilst rhIL-2 was purchased from PeproTech (Rocky Hill, NJ, USA).

### 4.2. Ethics Statement

The procurement of human blood and all experimental procedures were approved by the Human Research Ethics Committee of the Women’s and Children’s Health Network (WCHN), Adelaide, South Australia, in accordance to The National Statement on Ethical Conduct in Human Research (2007, updated 2018) (National Health and Medical Research Council Act 1992). Venous blood was collected from healthy adult volunteers with their informed consent and umbilical CB from healthy neonates with informed consent from pregnant women undergoing elective caesarean section.

### 4.3. Preparation of Cord Blood and Peripheral Blood Mononuclear Cells

Mononuclear cells (MC) were isolated from cord blood (CB) and adult peripheral blood (PB) by centrifugation on Ficoll^®^ Paque Plus media (GE Healthcare, Uppsala, Sweden) according to the manufacturer’s protocol. The interphase layer containing MC was harvested and then washed in RPMI-1640 medium supplemented with 2 mmol/L L-glutamine, 100 U/mL penicillin, 100 μg/mL streptomycin and 10% foetal calf serum (complete media). The CBMC or PBMC were cryopreserved at 5–10 × 10^6^ in 90% FCS and 10% DMSO, as previously described, for later functional analysis [32].

### 4.4. Isolation of T Cells

T cell isolation was performed using EasySep™ Human CD3^+^ T cell Isolation Kit (Stem Cell Technologies, Vancouver, Canada) by negative isolation, according to manufacturer protocols. Cryopreserved CBMC were rapidly thawed in a 37 °C water bath and washed in Phosphate-buffered saline (PBS) supplemented with 2%FCS and 1 mM EDTA (separation buffer). Viability was assessed by Trypan blue dye exclusion assay and found to be approximately 90%. Cells were resuspended in 0.25 to 2 mL of separation buffer, in 5 mL (12 × 75 mm) polystyrene round-bottom tubes (Corning, Cat #352058), maintaining a cell concentration of 5 × 10^7^ cells/mL. EasySep™ Human CD3^+^ T Cell Isolation Cocktail was added at 50 μL/mL of sample and incubated for 5 min. EasySep™ Dextran Rapid Spheres™ (50 μL/mL) were added and samples were reconstituted to 2.5 mL separation buffer. The T cells were retrieved using an EasySep™ Magnet (STEMCELL Technologies, Cat #18000), with purity consistently greater than 97%.

### 4.5. CBTC Maturation

Cord blood T cells were matured using an anti-CD3 and anti-CD28 co-stimulation method. Briefly, anti-CD3 antibodies (OKT3, Abcam, Cambridge, UK) were immobilised in 24 well plates by adding 2.5 μg/mL in Hank’s Balanced Salt Solution (HBSS) into each well, and refrigerating overnight or incubated for 3 h at 37 °C, and then washed with HBSS. At initiation of culture, anti-CD28 antibodies (Clone CD28.2, eBiosciences, San Diego, CA, USA) were added to a final concentration of 1 µg/mL with 1 × 10^6^ CBTC to each well in a total volume of 1 mL. After three days of culture, cells were counted and reseeded at 1 × 10^6^/mL with the addition of rhIL-2 (10 ng/mL), and this process was repeated on day 5. On day 7 the CD45RA/RO surface expression was measured by flow cytometry.

### 4.6. Apoptosis and Cell Viability Assays

Early and late apoptosis was ascertained using Annexin V and 7-AAD, respectively. The staining panel is described in Table 1. At the indicated time points 2 × 10^5^ cells were harvested and washed in 1 mL of Annexin V Binding buffer (10 mM HEPES (pH 7.4), 0.14 M NaCl, 0.25 mM CaCl_2_ and 0.5% BSA), incubated with Fluorescein isothiocyanate (FITC)-conjugated Annexin V for 20 min at room temperature (RT) in the dark. After two washes with Annexin V Binding buffer, samples were stained with 7-AAD for 5 min at RT and analysed by flow cytometry.

### 4.7. T Cell Proliferation Assays

*Radiometric method*: Briefly, in 96-well round bottom plates, T cells were added at 2 × 10^5^/well in RPMI/2.5% AB serum with or without PHA (2 μg/mL) or PMA (10 ng/mL) in triplicate, and incubated at 37 °C, 95% humidity and 5% CO_2_ for 72 h. Cells were pulsed with 1 μCi [^3^H]-thymidine 6 h prior to harvesting onto glass filter paper using a Titertek cell harvester. These were then added into vials with liquid scintillation cocktail (PerkinElmer) and processed in a β-scintilation counter (Wallac 1409 Liquid Scintillation Counter, PerkinElmer). Data are presented as a stimulation index (SI) of disintegrations per minute (DPM) in stimulated/DPM in unstimulated.

*Carboxyfluorescein succinimidyl ester (CFSE) based cell division*: T cells at 2 × 10^6^/mL were labelled with 1 µM of CFSE (CellTrace™ CFSE Cell Proliferation Kit, Invitrogen, Cat# C34554) in PBS without FCS. Following a 5 min incubation in the dark, the cells were washed with complete media and resuspended in X-VIVO 15 medium supplemented with 2 mmol/L L-glutamine, 100 U/mL penicillin, 100 μg/mL streptomycin and 10% FCS (complete X-VIVO media). Viability was examined by trypan blue dye exclusion. Proliferation was examined in T cells treated without or with anti-CD3/-CD28 antibodies in 96 well plates at 2 × 10^5^/well (as in Section 4.5), in complete X-VIVO/AB serum media. Cells were assessed on day three on a BD FACSCanto, using the FL-1/FITC channel to detect reduction in CFSE with cell division.

### 4.8. T Cell Maturation

To ascertain T cell maturation, CD45RA and CD45RO expression was examined at various time-points of T cell culture. For each time-point, 2 × 10^5^ cells were harvested and resuspended in 50 μL PBS/1% FCS (wash solution), followed by incubating with a cell surface staining antibody panel (as described in Table 2) for 20 min at RT in the dark. Following two washes in wash solution, the samples were acquired on a BD FACSCanto and analysed using FlowJo v10.1 (Ashland, OR, USA). Lymphocytes were gated based on their high CD45 expression and were further gated on CD3^+^ T cells to examine surface expression of CD45RA and CD45RO.

### 4.9. Detection of Intracellular Cytokines

Intracellular cytokines were measured in stimulated mature or immature T cells, using the BD Cytofix/Cytoperm™ Plus Permeabilization Kit with GolgiPlug. Briefly, 1 × 10^6^ cells/mL in RPMI/2.5% AB serum were stimulated with 50 nM PMA and 2 μg/mL PHA in the presence of Brefeldin A, and incubated at 37 °C/5%CO_2_ for 16–20 h. Cells were then washed in wash solution and resuspended in 200 µL of wash solution for surface staining with anti-CD3 PE-Cy5 (HIT3a), and anti-CD45 APC-H7 (2D1), for 15 to 20 min at RT in the dark. The cells were stained with the BD Horizon™ Fixable Viability Stain 510 (FVS510) Stock Solution (1:1000) in sodium azide- and protein-free PBS. At the end of 15 min incubation, cells were washed twice with 2 mL of wash solution. The cells were then fixed with 250 µL BD Cytofix/CytoPerm™ Fixation and Permeabilization Solution for 20 min at RT in the dark, and then permeabilized with 1 mL of BD Perm/Wash for 10 min at RT in the dark. A selected panel of antibodies for intracellular cytokine detection was then added to the cells (see Table 3, Table 4 and Table 5) and then incubated for 30 min at RT in the dark. After two washes with BD Perm/Wash, the samples were acquired on either a FACSCanto or FACSCanto II, as appropriate. Data analysis was performed using FlowJo v10.1. Cytokine expression and percentage positive cells were analysed after exclusion of doublets and dead cells by gating on FVS510^−^cell population.

### 4.10. Statistical Analysis

Statistical comparisons were performed using one-way ANOVA with post hoc Tukey’s multiple comparisons test, or Student’s *t*-testing. Correlations were performed using the two-tailed Pearson correlation coefficient. All statistical analyses were performed using Graphpad Prism v9 (GraphPad Software, La Jolla, CA, USA). A *p*-value of <0.05 was considered statistically significant for all analyses. 

## Figures and Tables

**Table 1:** Staining panel for apoptotic or dead cells.

Target/Antibody	Fluorochrome	Clone	Catalogue	Company
Annexin V	FITC		556420	BD
Anti-CD3	APC-CY7	SK7(Leu-4)	557832	BD
-	7-AAD		51-68981E	BD

**Table 2:** Staining panel for naïve/memory subsets in human CBTC.

Antibody	Fluorochrome	Clone	Catalogue	Company
Anti-CD45RA	FITC	HI100	555488	BD
Anti-CD45RO	PE	UCHL1	555493	BD
Anti-CD3	PE-CY5	HIT3a	555341	BD
Anti-CD45	APC-H7	2D1	641399	BD

**Table 3:** Intracellular staining of IL-4 and IFN-γ cytokines in T cells.

Antibody	Fluorochrome	Clone	Catalogue	Company
Anti-IFN-γ	FITC	4S.B3	554551	BD
Anti-IL-4	PE	8D4-8	12-7049-42	eBioscience
Anti-CD3	PE-CY5	HIT3a	555341	BD
Anti-CD45	APC-H7	2D1	641399	BD
Mouse-IgG1k	FITC	MOPC-21	555748	BD
Mouse-IgG1k	PE	MOPC-21	556650	BD

**Table 4:** Intracellular cytokines cocktail # 1 for T helper subsets.

Antibody	Fluorochrome	Clone	Catalogue	Company
Anti-IL-2	BV421	5344.111	562914	BD
Anti-IL-10	AF488	JES3-9D7	501413	BioLegend
Anti-LT-α	PE	359-81-11	554556	BD
Anti-IL-17A	PerCP-Cy™5.5	N49-653	560799	BD
Anti-TGF-βI	PE/Cy7	TW4-2F8	349610	BioLegend
Anti-TNF	APC	MAb11	554514	BD
Anti-IFN-γ	APC/CY7	4S.B3	502530	BioLegend
	BV510(viability stain)	-	564406	BD

**Table 5:** Intracellular cytokines cocktail # 2 for T helper subsets.

Target	Fluorochrome	Clone	Catalogue	Company
Anti-IL-13	BV421	JES10-5A2	563580	BD
Anti-IL-4	FITC	MP4-25D2	562047	BD
Anti-IL-21	PE	3A3-N2.1	562042	BD
Anti-IL-9	PerCP-Cy™5.5	MH9A3	561461	BD
Anti-IL-5	APC	TRFK5	562048	BD
Anti-IL-22	PE/Cy7	2G12A41	366707	BioLegend
Anti-IFN-γ	APC/CY7	4S.B3	502530	BioLegend
	BV510 (viability stain)	-	564406	BD

## Data Availability

The datasets generated during and analysed during the current study are available from the corresponding author on reasonable request.

## Supplementary Materials

Supplementary Materials can be found at https://www.mdpi.com/article/10.3390/ijms22094907/s1.

## Abbreviations

CBTC	cord blood T cells
CBMC	CB mononuclear cells
PBMC	peripheral blood MC
AB	adult blood
PKC	Protein Kinase C
PHA	Phytohaemagglutinin
PMA	Phorbol myristate acetate
IFN-γ	Interferon-gamma
rhIL-2	recombinant human interleukin 2
TNF	tumour necrosis factor-alpha
LT-α	Lymphotoxin α
TGF-β	Transforming growth factor-beta
CFSE	carboxyfluorescein succinimidyl ester
MFI	Median Fluorescence Intensity
[^3^H]-TdR	tritiated thymidine
7-AAD	7-aminoactinomycin D
IgE	Immunoglobulin E
FcεR1	High-affinity IgE receptor
RPMI 1640	Roswell Park Memorial Institute 1640
IL-5R	IL-5 receptor
FCS	foetal calf serum
