# Supplementary material for: Cord Blood T Cells Expressing High and Low PKCζ Levels Develop into Cells with a Propensity to Display Th1 and Th9 Cytokine Profiles, Respectively
Source: Int J Mol Sci. 2021 May 5;22(9):4907. doi: 10.3390/ijms22094907 (PMC8124775; doi:10.3390/ijms22094907)
Supplement: Supplementary file 1 [file ijms-22-04907-s001.zip › ijms-1183517-supplementary.pdf]

International Journal of
Molecular Sciences

MDPI

Article

Cord Blood T Cells Expressing High and Low PKCζ Levels Develop into Cells with a Propensity to Display Th1 and Th9 Cytokine Profiles, Respectively

Khalida Perveen [1,2], Alex Quach [1,2], Andrew McPhee [4], Susan L. Prescott [5,6], Simon C. Barry [2], Charles S. Hii [1,2] and Antonio Ferrante [1,2,3,*]

* Correspondence: Antonio.Ferrante@ade;laide.edu.au

This PDF file includes:

Figures S1 to S4

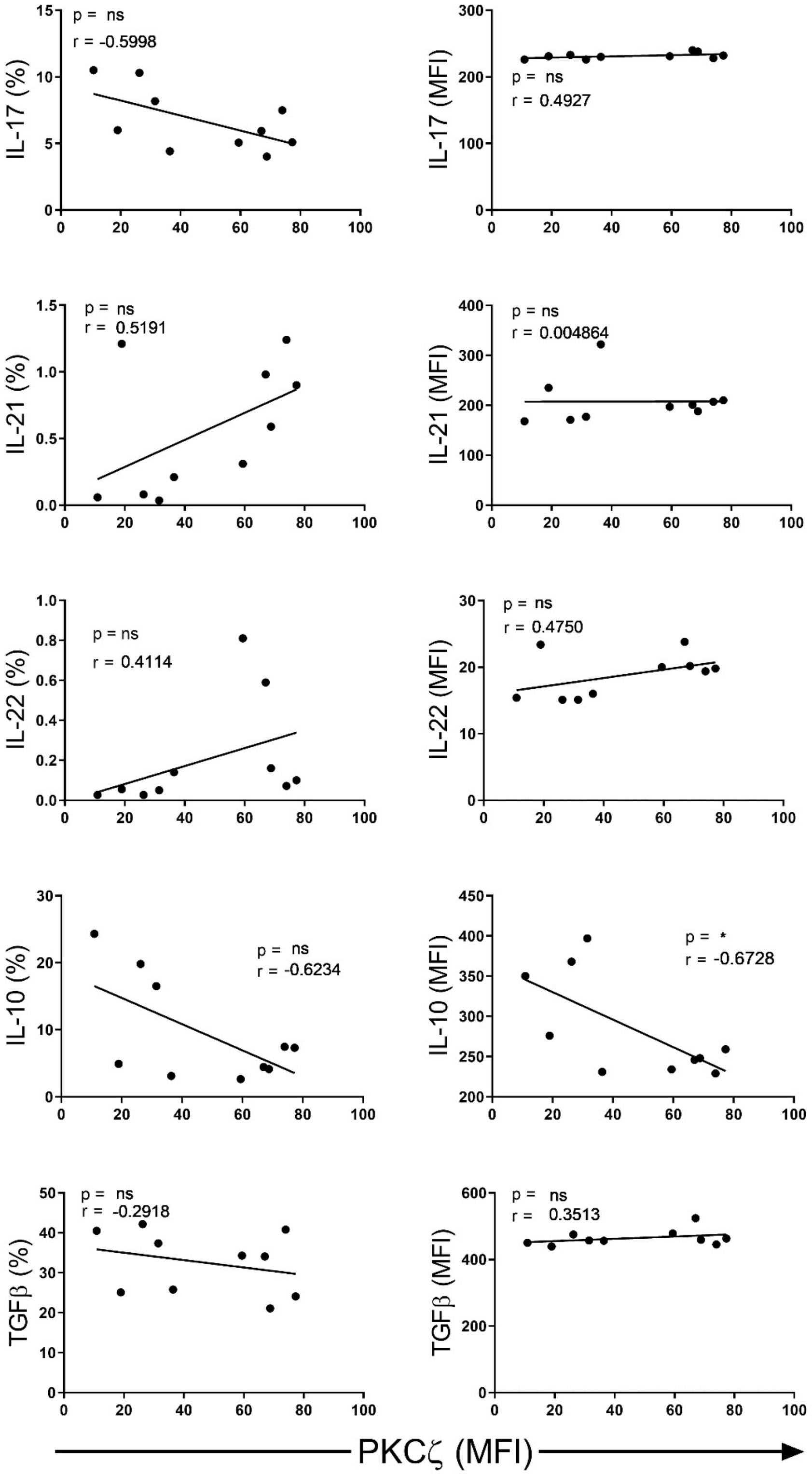

Figure S1. Correlation of PKCζ expression with other T cell cytokines. Data from figure 3 and 4 were subjected to correlation analysis Table 3. T cells for each cytokine) and the second column represents the MFI of the gated positive respective cytokine. ns: not significant, * $P < 0.05$. Correlations were performed using the two-tailed Pearson correlation coefficient.

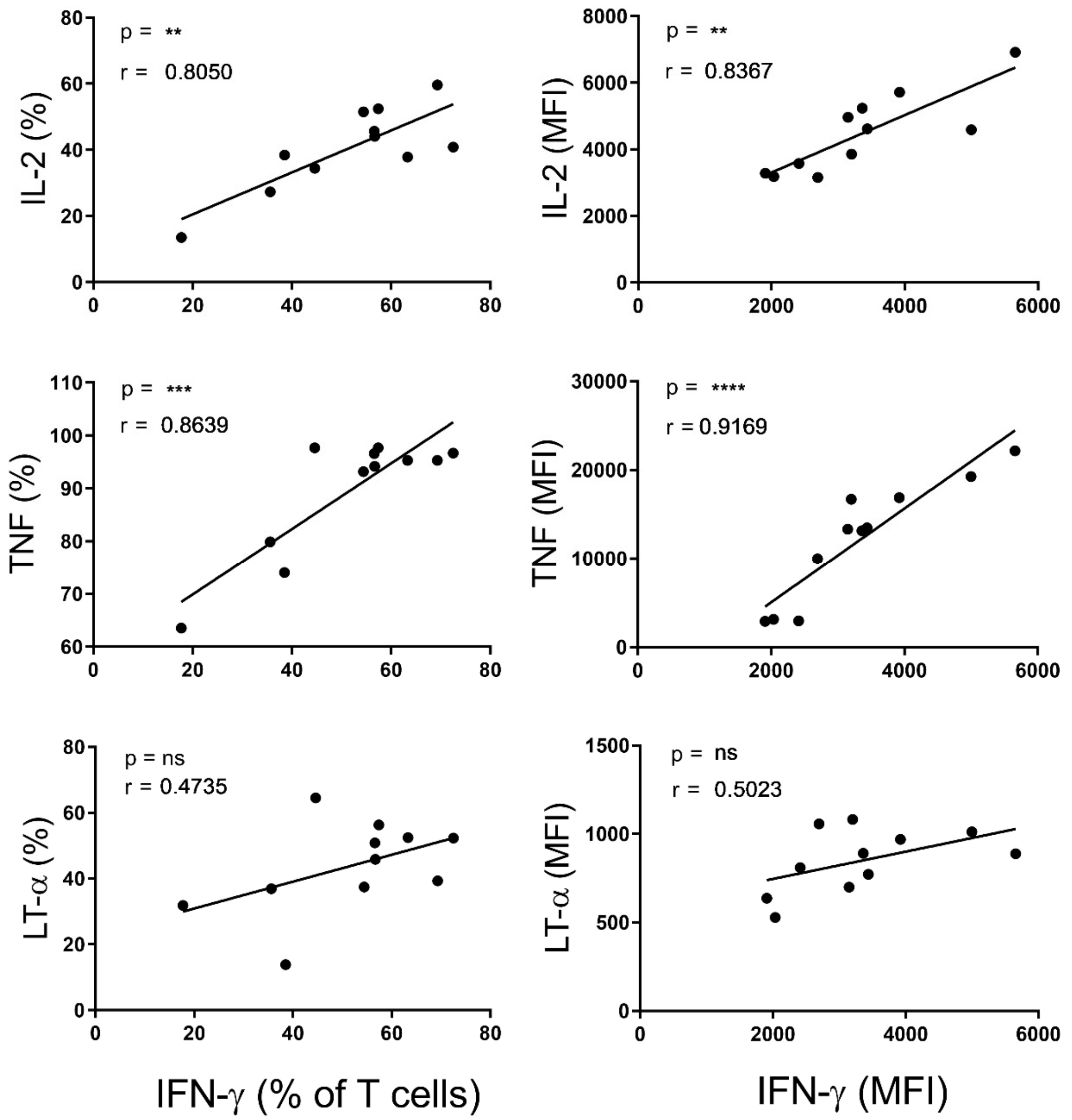

Figure S2. Correlation of IFN-γ with other Th1 cytokines. Data from figure 3 were subjected to correlation analysis The first column represents the Pearson correlation of IFN-γ with representative cytokines (percentage of $CD3^+$ T cells for each cytokine) and the second column represents the MFI of the gated positive respective cytokine. ns: not significant, ** $P < 0.01$, *** $P < 0.001$, ****: $P < 0.0001$. Correlations were performed using the two-tailed Pearson correlation coefficient.

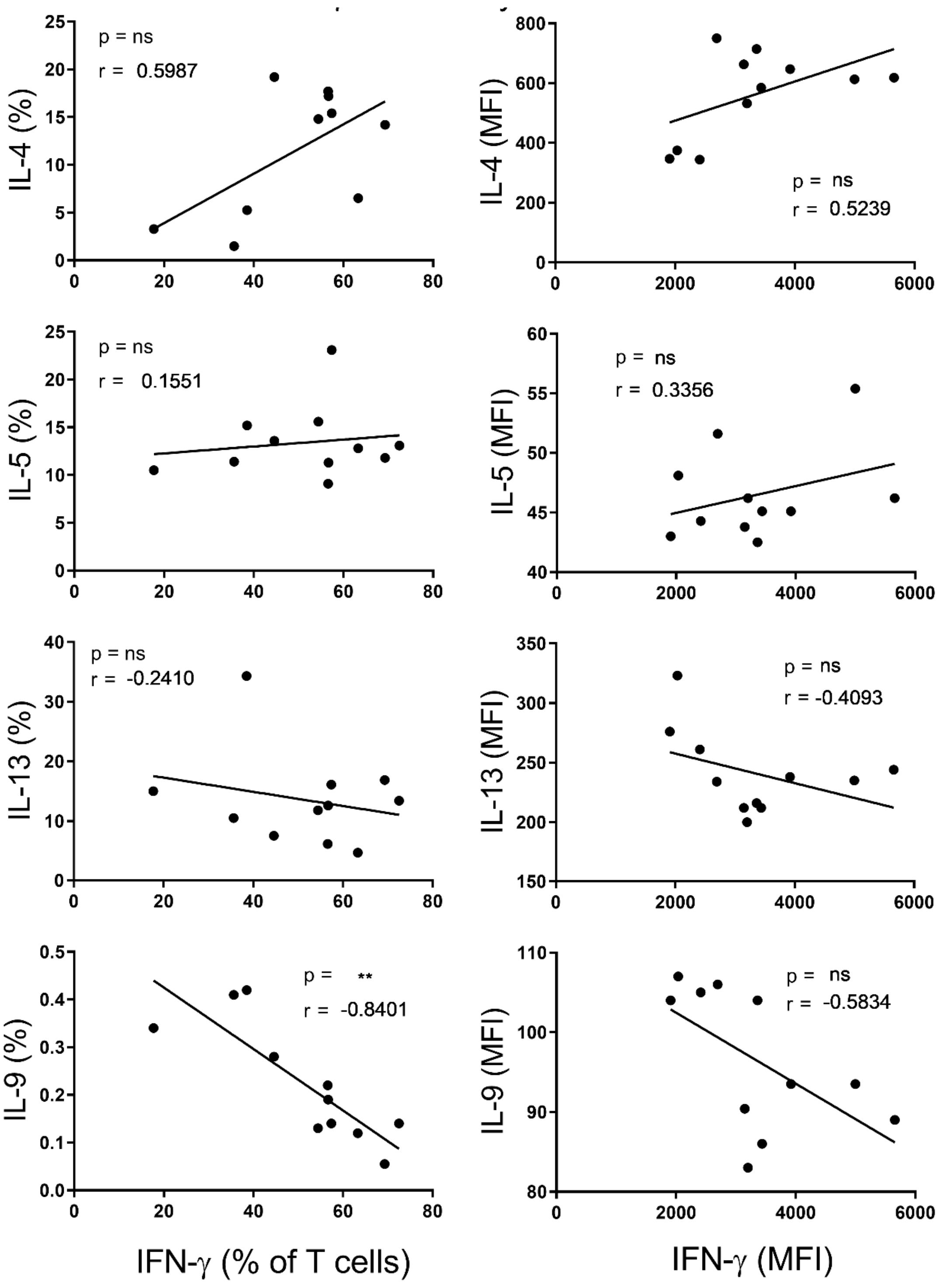

Figure S3. Correlation of IFN-γ with Th2 cytokines. Data from figure 3 and 4 were subjected to correlation analysis The first column represents the Pearson correlation of IFN-γ with representative cytokines (percentage of CD3+ T cells for each cytokine) and the second column represents the MFI of the gated positive respective cytokine. ns: not significant, ** *P* < 0.01. Correlations were performed using the two-tailed Pearson correlation coefficient.

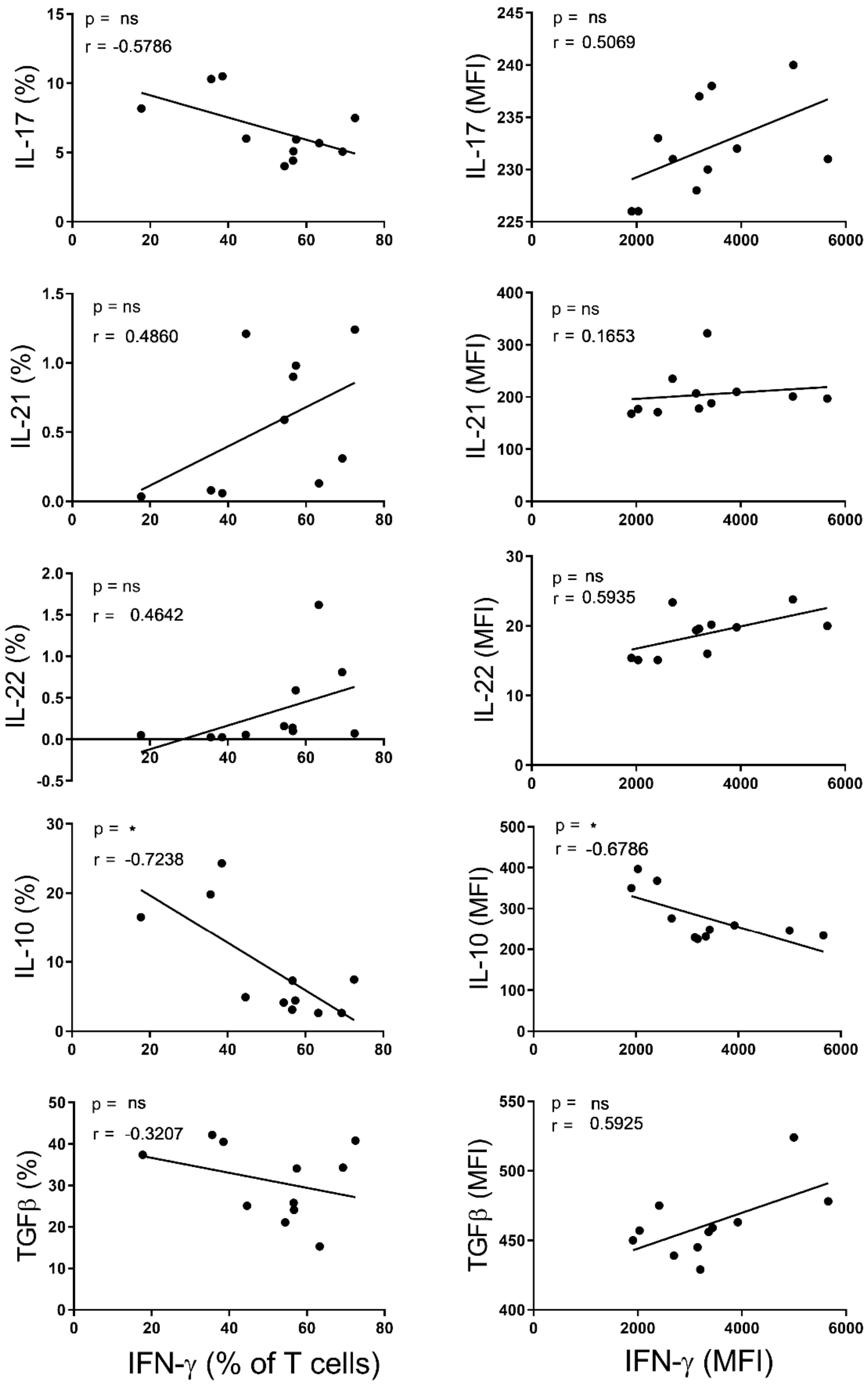

Figure S4. Correlation of IFN-γ with other cytokines. Data from figure 3 and 4 were subjected to correlation analysis. The first column represents the Pearson correlation of IFN-γ with representative cytokines (percentage of $CD3^+$ T cells for each cytokine) and the second column represents the MFI of the gated positive respective cytokine. ns: not significant, * $P < 0.05$. Correlations were performed using the two-tailed Pearson correlation coefficient.
